# Supplementary material for: Optional Vaccines in Children—Knowledge, Attitudes, and Practices in Romanian Parents
Source: Vaccines (Basel). 2022 Mar 7;10(3):404. doi: 10.3390/vaccines10030404 (PMC8955643; doi:10.3390/vaccines10030404)
Supplement: Supplementary file 1 [file vaccines-10-00404-s001.zip › vaccines-1612159-supplementary.pdf]

**Supplementary material: Questionnaire**

**General data**

1. Sex of parent
   male
   female
2. Age of parent (in years)
   *... free response*
3. Last school graduated:
   unfinished general school / without studies
   completed general school
   vocational school
   high school
   post-high school
   university studies
3. Do you live in the city or the village?
   In the city
   In the village
4. How many children do you have?
   1
   2
   3
   4
   5 or more
5. Age of child 1 - first child born (in completed years):
   *...free response*
6. Age of child 2 (in completed years):
   *... free response*
7. Age of child 3 (in completed years):
   *... free response*
8. Ages of other children (in completed years), if you have more than 4 children, list their ages
   *... free response*

**Data on parents' general knowledge about vaccination**

9. Which of the following childhood diseases can be prevented by vaccination? (You can select more than one answer)
   Scarlet fever
   Measles
   Streptococcal pharyngitis
   Whooping cough
   Meningococcal meningitis
   Hepatitis B
   Pneumococcal pneumonia
   Hepatitis C
   Chickenpox

10. Which of the following vaccines are part of the free vaccination scheme? (You can select more than one answer)

influenza vaccine
hepatitis B vaccine
varicella vaccine
meningococcal meningitis vaccine
streptococcal vaccine
measles vaccine
tetanus vaccine
diphtheria vaccine

11. Have you heard of additional vaccines that can be given to your child/children?

Yes
No

Data on childhood vaccination and parental perception

12. What are the main sources of information you use to inform yourself about vaccines for your child/children? (You can select more than one answer)

Media (TV, radio, etc.)
Internet (social networks, blogs)
Family doctor
Pediatrician
Medical books/magazines
Family
Discussions with other parents

13. Have you or your spouse/partner ever discussed with your family doctor/pediatrician about the vaccines offered free of charge by the Ministry of Health for your child/children?

Yes, with the family doctor
Yes, with the pediatrician
Yes, and with both the family doctor and the pediatrician
No

14. Have you or your spouse/partner ever discussed with your family doctor/pediatrician about optional vaccines for your child/children?

Yes, with the family doctor
Yes, with the pediatrician
Yes, and with both the family doctor and the pediatrician
No

15. Do you think the family doctor or pediatrician should be more involved in discussions with parents about optional vaccination?

Yes
No

16. Has your child(ren) been fully vaccinated with the vaccines provided free of charge by the Ministry of Health up to the current age of your child(ren)?

Yes, completely
Yes, partially
No
I don't know

17. If you answered "Yes, partially" or "No" to the previous question, which vaccines did you not have?

BCG (against tuberculosis)
Anti-hepatitis B
Hexavalent
Anti-pneumococcal (Prevenar)
MMR (measles-mumps-rubella)
Diphtheria-tetanus-pertussis

18. If you answered "Yes, partially" or "No" to the previous question, what was the reason for not getting those vaccines?
I've been putting it off, but I intend to do it
I was afraid of potential side effects
My family doctor advised me not to do it
My pediatrician advised me not to do it

19. Have you administered at least one additional vaccine to at least one of your children?
Yes
No

20. If you answered "No" to the previous question, what are the reasons why you did not administer any additional vaccine to your child/children?
I was not aware of the existence of the additional vaccines
My family doctor/pediatrician advised me not to have any additional vaccines
I was afraid of potential side effects
The high costs of the optional vaccination
I encountered difficulties in purchasing as stocks were unavailable
Other *(... free response)*

21. If you answered "YES" to the previous question, which vaccines did you additionally administer to your first child (child 1)?
rotavirus vaccine
varicella vaccine
pneumococcal vaccine (Prevenar)
ACYW meningococcal vaccine (Nimenrix)
meningococcal B vaccine (Bexsero/Trumenba)
intranasal influenza vaccine
injectable influenza vaccine
hepatitis A vaccine
I do not know exactly; the child's vaccination was carried out by the spouse/partner/partner

22. If you answered YES to the previous question, which vaccines did you additionally administer to your second child (child 2)?
rotavirus vaccine
varicella vaccine
pneumococcal vaccine (Prevenar)
ACYW meningococcal vaccine (Nimenrix)
meningococcal B vaccine (Bexsero/Trumenba)
intranasal influenza vaccine
injectable influenza vaccine
hepatitis A vaccine
I have not administered my second child any optional vaccines at the moment, but I intend to do so soon.

I don't know exactly; the child's vaccination was done by the spouse/partner/partner

23. If you answered YES to the previous question, which vaccines did you additionally administer to your third child (child 3)?

rotavirus vaccine
varicella vaccine
pneumococcal vaccine (Prevenar)
ACYW meningococcal vaccine (Nimenrix)
meningococcal B vaccine (Bexsero/Trumenba)
intranasal influenza vaccine
injectable influenza vaccine
hepatitis A vaccine
I have not administered any optional vaccines to my third child at the moment, but intend to do so soon

24. If you answered YES to the previous question and you have more than 4 children, please write below what additional vaccinations children 4, 5 and so on have had.

*free response....*

25. Do you consider that optional vaccination is additional health safety for your child/children?

Yes
No

26. Do you consider that some of the optional vaccines should be included in the standard vaccination scheme and offered free of charge by the Ministry of Health?

Yes
No

27. If you answered "Yes" to the previous question, in your opinion and based on your knowledge, what are the 3 most important vaccines that should be included in the standard, free vaccination schedule? (choose 3 answers)

rotavirus vaccine
varicella vaccine
ACYW meningococcal vaccine (Nimenrix)
meningococcal B vaccine (Bexsero/Trumenba)
intranasal influenza vaccine
injectable influenza vaccine
hepatitis A vaccine

29. Which of the infections prevented by optional vaccination frightens you the most?

infection with influenza viruses (flu)
varicella virus infection (chickenpox / chickenpox)
rotavirus infection (rotavirus enterocolitis)
meningococcal infection (meningococcal meningitis)
hepatitis A virus infection (hepatitis A)
none
